# Interleukins 17 and 23 in patients with gastric neoplasms

**DOI:** 10.1038/srep37451

**Published:** 2016-11-21

**Authors:** Wojciech Błogowski, Anna Madej-Michniewicz, Natalia Marczuk, Barbara Dołęgowska, Teresa Starzyńska

**Affiliations:** 1 Department of Internal Medicine, University of Zielona Gora, Zielona Gora, Poland; 2 Department of Gastroenterology, Pomeranian Medical University, Szczecin, Poland; 3 Department of Microbiology, Immunology and Laboratory Medicine, Pomeranian Medical University, Szczecin, Poland

## Abstract

Recently there has been heightened interest in the potential significance of interleukin (IL)-17 and IL-23 in the development/progression of human malignancies. Here, we analyzed the systemic levels of these cytokines in 75 patients with different types of gastric neoplasms (carcinoma, gastrointestinal stromal tumors, neuroendocrine neoplasms, and lymphomas) and 42 healthy volunteers. We found that patients with all types of gastric neoplasms have significantly lower IL-23 levels. However, in comparison to the levels in healthy individuals, IL-17 concentrations were lower only in patients with types of gastric neoplasms other than carcinoma. Interestingly, IL-17 levels significantly differed between patients with early and advanced gastric carcinoma. No significant associations were detected between the systemic levels of examined interleukins and TNM staging. However, peripheral levels of IL-23 were correlated with the absolute numbers of circulating populations of bone marrow-derived mesenchymal and very small embryonic/epiblast-like stem cells in patients with gastric carcinoma. ROC curve analyses demonstrated that systemic levels of IL-17 seem to meet basic criteria for consideration as a helpful diagnostic marker in the detection of gastric carcinoma. In conclusion, our study provides translational evidence confirming the clinical significance of IL-17 and IL-23 in the pathogenesis of different types of gastric neoplasms in humans.

Malignancies originating from the stomach represent an important diagnostic and therapeutic challenge in modern clinical gastroenterology^1^,^2^. In the vast majority of cases, patients in whom gastric neoplasms are detected are diagnosed with gastric carcinoma. Unfortunately, unless this disease is detected and successfully treated at its earliest stages, it is associated with a generally poor prognosis^1^,^3^. Other less frequently occurring types of neoplasms, which may have milder and less debilitating clinical outcomes, may also develop within the gastric tissue. These include gastrointestinal stromal tumors (GISTs), neuroendocrine neoplasms (NENs), and/or diverse types of gastric lymphomas^4^,^5^,^6^,^7^,^8^,^9^. However, even though significant effort has been directed toward elucidating the pathogenesis of various types of gastric neoplasms, the exact mechanisms and factors responsible for the development and/or progression of these tumors in humans remain unknown.

Cytokines are believed to play a significant role in the pathogenesis of multiple types of neoplasms, including those malignancies that originate from the gastrointestinal tract. In addition, they appear to hold promise as targets for anti-cancer therapy^9^,^10^,^11^,^12^,^13^. Among this vast group of biochemical molecules, interleukins (IL) are becoming the focus of increasing interest, because these substances may influence various molecular processes that are crucial for the successful development and spread of malignancies owing to their unique nature and function. Recently, it has been demonstrated that, among a group of “inflammation-related” neoplasms, including gastrointestinal malignancies, the action of two inflammatory cytokines, IL-17 and IL-23, may be of particular significance^14^,^15^,^16^. This concept is based on the fact that elevated tissue expression of IL-17 and IL-23 has been detected in colon cancer tissue samples, and this expression has been linked to outcomes in affected patients^17^. Moreover, in a series of studies performed in a CPC-APC mouse model of colon cancer, several researchers have demonstrated that manipulations in the biological activity of IL-17/IL-23 (gene disruption, receptor ablation, or neutralization using specific antibodies) inhibit colonic tumor development due to decreases in cell proliferation, as well as preventing progression from adenoma to carcinoma^18^,^19^,^20^. Unfortunately, these observations have never been confirmed in clinical studies focused on comprehensive analysis of patients with different types of gastric neoplasms.

Taking all of these molecular observations into consideration, in the current study, we aimed to verify an original hypothesis that an abnormal balance in systemic levels of IL-17 and IL-23 exists in patients with different types of gastric neoplasms. This is associated with both the clinical presentation of gastric tumors and with the recently reported phenomenon of intensified peripheral trafficking of selected populations of bone marrow-derived stem cells (BMSCs) in patients with gastric carcinoma^21^. We additionally posited that measurements of systemic levels of examined interleukins could be of potential diagnostic value in differentiating gastric carcinoma from other types of gastric neoplasms in humans.

To achieve this goal, we aimed to: i) examine and compare IL-17 and IL-23 levels among groups of patients and healthy volunteers; ii) verify the potential associations between levels of examined interleukins and both clinical staging of gastric carcinoma (evaluated according to the Tumor Node Metastasis [TNM] classification) and absolute numbers of different populations of circulating BMSCs; and iii) estimate the preliminary clinical value that can be derived from measurements of systemic levels of IL-17 and IL-23 in patients with lesions detected within the gastric tissue, as novel diagnostic serum markers of gastric carcinoma in humans.

## Results

### Statistical comparison of included patients

Comprehensive evaluation revealed no statistically significant differences in anthropometric and laboratory parameters between the analyzed groups of recruited individuals (Table 1). While patients with gastric neoplasms seemed to have slightly lower body mass index (BMI) values and hemoglobin levels than control individuals, these differences did not reach statistical significance (p = 0.09 and p = 0.07 for BMI and hemoglobin, respectively). No statistically significant differences were observed between patients with gastric carcinoma and those with other types of gastric neoplasms.

### Comparison of systemic levels of examined interleukins in patients with gastric carcinoma, other types of gastric neoplasms, and control individuals

**Figure 1:**
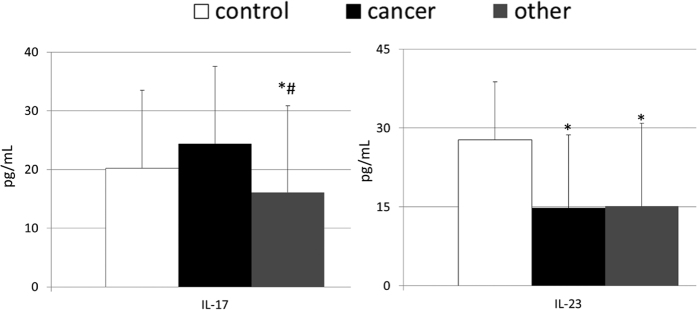
Levels of examined interleukins in patients with gastric carcinoma, other gastric neoplasms and control individuals together with their statistical comparison (means ± standard deviation). IL – interleukin. *p < 0.01 – level of significance (vs “control” group). ^#^p < 0.05 – level of significance (vs “cancer” group).

The mean peripheral levels of examined interleukins in patients with gastric carcinoma, other gastric neoplasms, and healthy individuals are presented in Fig. 1. We found that IL-17 levels were not significantly different between patients with gastric carcinoma and healthy individuals (p = 0.07). However, significantly lower concentrations of this cytokine were observed in patients diagnosed with other types of gastric neoplasms than in both healthy individuals and those with gastric carcinoma. Interestingly, both groups of patients diagnosed with gastric malignancies had significantly (approximately 50%) lower IL-23 concentrations than healthy individuals. In addition, we observed no significant differences in the mean values of this cytokine between patients with gastric carcinomas and those with other types of gastric malignancies. When we compared mean levels of the interleukins we examined among patients with GISTs, NENs, and lymphomas, we did not observe any statistically significant differences (*unpublished observation*).

### Evaluation of associations between interleukins and circulating bone marrow-derived stem cells

Next, we attempted to determine whether levels of the interleukins we examined here were associated with concentrations of other interleukins (IL-1, IL-6, IL-8, IL-10, and IL-12) and the values of their respective ratios that we recently reported^22^. We found that, in patients with gastric carcinoma, peripheral levels of IL-17 were correlated only with IL-12 concentrations, whereas IL-23 levels were significantly associated with IL-10 values (Table 2). We also performed an analysis of correlations between the examined interleukins and the absolute numbers of circulating BMSCs reported previously^21^. This analysis demonstrated that systemic levels of IL-23 were negatively correlated with selected populations of BMSCs, mainly very small embryonic/epiblast-like stem cells and mesenchymal stem cells (Table 2).

### Evaluation of clinical associations between examined cytokines and gastric carcinoma

**Figure 2:**
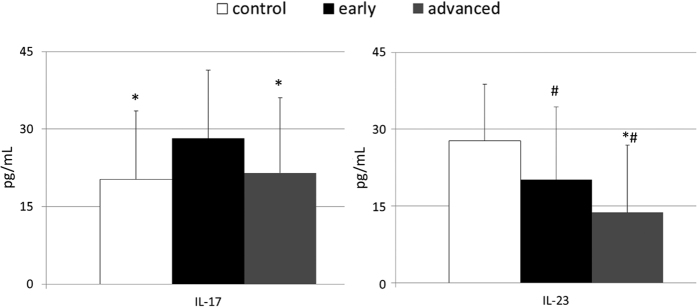
Mean values of examined interleukins’ levels in patients with early and advanced gastric carcinoma, as well as in control individuals together with their statistical comparison (means ± standard deviation). IL–interleukin. *p < 0.01 – level of significance (vs “early” group). ^#^p < 0.01 – level of significance (vs “control” group).

To determine whether the observed alterations in systemic levels of the interleukins we examined were associated with the clinical presentation of gastric carcinoma among our patients, we performed a statistical comparison of levels of these cytokines in individuals with cancer subdivided into two groups, those with early and advanced gastric carcinoma defined according to the Japanese criteria. These analyses revealed that, in comparison to healthy individuals, patients with early gastric carcinoma had significantly higher mean values of IL-17 and lower mean values of IL-23, whereas individuals with advanced gastric carcinoma had mean IL-17 concentrations comparable to those observed in healthy individuals and IL-23 levels lower than those observed in both healthy controls and patients with early gastric carcinoma (Fig. 2). We also performed multivariate regression analyses to verify whether systemic levels of the examined interleukins were associated with clinical staging of gastric carcinoma, established according to the TNM classification. Our results showed that peripheral levels of the examined interleukins were not significantly associated with TNM staging of gastric carcinoma in our patient sample (Table 3).

### Clinical diagnostic value of examined interleukins

**Figure 3:**
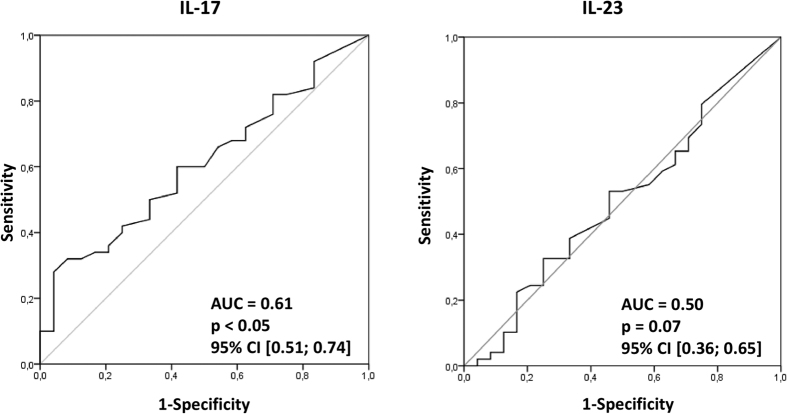
Receiver operating characteristics (ROC) curves of examined interleukins as indicators of gastric carcinoma. Calculated sensitivity (y-axis) is plotted against 1-specificity formula (x-axis) for examined interleukins - IL-17 and IL-23. IL–interleukin, AUC–area under curve, p–level of significance, CI–confidence interval.

Finally, we aimed to verify, at least on a preliminary basis, the potential diagnostic value of the examined interleukins for the detection of gastric carcinoma in humans. To determine whether systemic levels of IL-17 and IL-23 could serve as novel markers of gastric carcinoma, we constructed receiver operating characteristic curves, and determined the approximate area under the curve values to assess the suitability of these cytokines as potential novel diagnostic markers (Fig. 3). Our analysis demonstrated that only levels of IL-17 seem to meet basic criteria for potential consideration as a novel candidate marker for the detection of gastric carcinoma in humans.

## Discussion

It has been noted for several years that interaction between various cytokines may play a significant role in the development and/or progression of malignancies. Recently, much attention has focused on the action of the IL-17/IL-23 axis, which is believed to be a major player in inflammation-related malignancies, such as gastric neoplasms^9^,^10^,^11^,^12^,^13^,^14^,^15^,^16^,^22^,^23^. Indeed, epidemiological analyses revealed that Asian patients with selected polymorphisms in genes coding for both IL-17 and IL-23 may have an altered risk for developing gastric cancer^24^,^25^,^26^,^27^,^28^,^29^. However, very little is known at present about the clinical significance of IL-17 and IL-23 in the pathogenesis of different types of gastric neoplasms in humans. Therefore, in the present study, we aimed to comprehensively evaluate levels of these cytokines. We verified their associations with the clinical presentation of gastric tumors and the phenomenon of intensified peripheral trafficking of BMSCs, which has been recently reported in patients with gastric carcinoma^21^. We also focused on preliminary estimation of the diagnostic value of these cytokines for detection and/or differentiation of gastric carcinoma from other types of gastric neoplasms in humans.

In this study, we found that in comparison to healthy individuals, patients with gastric neoplasms had altered IL-17 and IL-23 levels. Namely, in the general group of patients with gastric carcinoma, mean concentrations of IL-17 were statistically comparable to those observed in healthy controls, findings similar to those of the study by Malek-Hosseini *et al*.^30^. However, several authors have previously demonstrated that elevated systemic levels of this cytokine are present in patients with gastric carcinoma^31^,^32^,^33^,^34^,^35^. We believe that these discrepancies may be potentially explained by the different profiles of patients with gastric carcinoma included in the analyses. In the studies in which the authors observed significant increases in systemic IL-17 levels, the majority of the recruited patients with gastric carcinoma were diagnosed with early (I/II TNM) stages of the disease, while in our study as well as in the study by Malek-Hosseini *et al*.^30^, over 60% of patients presented with more advanced disease. The results of our study demonstrate that significantly elevated IL-17 levels are observed in patients with gastric carcinoma detected at an early stage, whereas systemic concentrations of this cytokine in individuals with advanced disease are comparable to the values observed in healthy controls. Therefore, the results of our study provide a potential explanation for previously reported discrepancies, and highlight the fact that the interpretation of results regarding IL-17 levels in patients with gastric carcinoma is strongly dependent on the profile of included patients, who should be carefully characterized and evaluated according to the Japanese criteria rather than staged solely based on the TNM classification. This observation seems to be of importance also in terms of analyses of IL-23 levels in patients with gastric carcinoma. In the current study, we found that levels of this cytokine significantly differed between subgroups of gastric carcinoma patients created on the basis of the Japanese classification criteria, but these differences were not associated with TNM staging. However, it is important to highlight that, in our study, concentrations of this molecule were significantly lower in patients with gastric carcinoma than in healthy individuals; however, Liu *et al*.^31^ reported a significant increase in IL-23 levels among their group of gastric carcinoma patients. It is difficult to definitively explain this discrepancy according to the current knowledge base, as there are no other reports available in the literature on this topic. We hypothesize that the discrepancy may be caused by multiple factors, including completely different ethnic origins of study populations. In previous studies, it has been demonstrated that the genetic expression of selected cytokines, their impact on gastric carcinoma risk, and their importance in the development/progression of this disease may vary among patients of different ethnic origin, and may also be influenced by the genetic profile of *Helicobacter pylori* strains present in different parts of the world^36^,^37^. In terms of the Polish population, our previous study regarding the significance of interleukins in the pathogenesis of pancreatic neoplasms also revealed significantly lower IL-23 concentrations in patients with pancreatic cancer^38^. Therefore, we believe that further clinical, experimental, and genetic studies are needed to fully define, understand, and explain the aforementioned discrepancies regarding IL-23 levels among patients with gastrointestinal cancers.

Unfortunately, in our clinical study we were unable to define the exact molecular mechanisms of action of the examined interleukins in patients with gastric carcinoma. While there are experimental studies demonstrating that IL-17-related stimulation/activity may not be essential for the successful development and/or progression of gastric cancers, it has been shown that these molecules may promote the invasiveness of gastric cancer cells through activation of the nuclear factor-κB (NF-κB) pathway and subsequent upregulation of the expression of metalloproteinases^39^,^40^. The results presented here suggest a potential novel significance of these cytokines in the pathogenesis of gastrointestinal neoplasms in humans. Namely, similar to the findings of our previous study, in which patients with pancreatic cancer were analyzed, in the current study, we found that IL-23 levels are significantly associated with absolute numbers of circulating mesenchymal and very small embryonic/epiblast-like stem cells in patients with gastric cancer^38^,^41^. To date, little is known about the impact of this cytokine on the homeostasis of the bone marrow environment itself or on circulating stem cells. In this study, we further confirmed that, in patients with different types of gastrointestinal malignancies, IL-23 does have a significance on this phenomenon, and we believe this subject should be granted attention in further clinical and experimental studies.

Moreover, it is interesting that, in comparison to the healthy individuals, the general group of patients with gastric neoplasms other than gastric carcinoma showed significantly altered profiles of the examined cytokines. Unfortunately, the direct biochemical mechanisms associated with the development and/or progression of these rare types of gastric neoplasms remain unknown. However, our study highlights that the action of the IL-17/IL-23 axis may be a common pathway of interactions involved in the development and/or progression of all types of gastric malignancies in humans. In our recent aforementioned study, based on analysis of patients with different types of pancreatic neoplasms, we observed similar results regarding the potential significance of IL-23, in particular, in the pathogenesis of these tumors in humans^38^. It is to be hoped that further clinical and experimental studies will be conducted in the future and will fully define the exact molecular roles of IL-17/IL-23 in the pathogenesis of different types of gastrointestinal malignancies in humans.

Finally, we also evaluated the potential diagnostic value of the examined interleukins for eventual use as novel biochemical indicators detecting and/or differentiating gastric carcinoma from other types of gastric lesions in humans. Our analyses revealed that only measurements of IL-17 may meet initial criteria for further consideration as a potential promising marker of gastric carcinoma in humans. However, from the analyses we conducted, it seems that this cytokine will definitively not be a suitable marker for independent decision-making, as its diagnostic value is less promising than those of various cytokines previously reported^22^,^42^,^43^, and it may be considered only as an additional helpful factor that could support conventional diagnostic processes.

In summary, our study supported the translational significance of IL-17 and IL-23 in the pathogenesis and clinical presentation of different types of gastric neoplasms in humans. Moreover, it highlights several associations between IL-23 and intensified BMSC trafficking in patients with gastric carcinoma. Finally, it demonstrates that systemic levels of the interleukins examined here do not possess sufficient diagnostic value to be used as independent markers for the detection of gastric carcinoma.

## Material and Methods

### Ethics statement

This study was performed in accordance with appropriate regulations and guidelines highlighted in the “World Medical Association Declaration of Helsinki – Ethical Principles for Medical Research Involving Human Subjects”. The study protocol was approved by the Institutional Bioethical Committee of the Pomeranian Medical University in Szczecin, and all patients provided written informed consent prior to inclusion in the study.

### Patients and blood samples

A total of 117 generally healthy individuals were included in the study protocol. These patients were divided into following groups: a “cancer” group of newly diagnosed gastric carcinoma patients (n = 50), an “other” malignancies group to which patients with gastrointestinal stromal tumors - GISTs (n = 5), gastric neuroendocrine neoplasms – NENs (n = 12) and primary gastric lymphomas (n = 8) were included, as well as a “control” group of healthy volunteers (n = 42).

Analogically as in our previous studies^21^,^22^ the final diagnosis of gastric neoplasm was based on biopsy specimen analysis. In order to establish disease staging, patients underwent ultrasonography, computed tomography and/or endoscopic ultrasonography, as well as chest x-ray examinations. In the “cancer” group 30 patients were diagnosed with intestinal, 12 with diffuse and 8 with mixed type of gastric carcinoma according to the Lauren’s classification. According to the Tumor Node Metastasis (TNM) classification, 19 patients had stage I gastric carcinoma, 3 stage II, 5 stage III and in 21 patients the disease presented with metastasis (stage IV). In 2 patients we were not able to evaluate the exact stage of the malignancy because they died before any further diagnostic/clinical assessment. Among our cancer patients 19 patients were diagnosed with early and 31 with advanced gastric cancer, according to the Japanese criteria. Histological analysis revealed following types of gastric cancer in our patients: *adenocarcinoma* (n = 34), *carcinoma mucocellulare* (n = 5), *signet ring cell carcinoma* (n = 2), *carcinoma male differentiatum* (n = 2), and *mixed type* (n = 7).

Among patients with GISTs, in 3 cases stage I low grade tumors were diagnosed, and in 2 patients the tumors were detected in stage II and III with high grade of malignant potential. In all cases GISTs were primarily localized in the fundus of the stomach. In case of patients with NEN lesions, all of the diagnosed tumors were non-functional, and were located in the fundus of the stomach. In 10 patients low grade malignancy tumors were observed (NEN G1), and in 2 cases NEN G2 neoplasms were diagnosed. None of the NEN patients presented any signs of metastasis neither to lymph nodes nor to distant solid organs. Patients suffering from primary gastric lymphomas presented following histological types: diffuse large B-cell lymphoma (n = 4), Burkitt lymphoma (n = 2), small lymphocyte lymphoma (n = 1) and mucus-associated lymphoid tissue lymphoma (n = 1). The general characteristics of the individuals enrolled in the study, together with a statistical comparison of these features between the examined groups, are presented in Table 1.

Peripheral blood samples (8–10 mL) were collected from all included individuals. Samples were processed immediately according to standard laboratory protocols, and plasma was separated, frozen, and stored at −80 °C until further assessment.

### Analysis of systemic levels of cytokines

The systemic concentrations of interleukins (IL-17 and IL-23) were measured using commercially available, high-sensitivity ELISA kits (*R&D Systems, Minneapolis, MN, USA*) according to the manufacturer instructions and recommendations.

### Statistical Methods

Analogically as in our previous studies^21^,^22^,^44^,^45^,^46^,^47^,^48^ the Shapiro–Wilk test was used to determine the distribution of the continuous variables analyzed. The Student’s t-test was used to compare mean parameter values between the examined groups (for normally distributed variables). For variables that were not normally distributed, the values were log transformed. If a normal distribution was then achieved, these transformed variables were also compared using the Student’s t-test. However, if the transformation did not result in a normal distribution, a Mann-Whitney U-test was performed. Correlations between various analyzed parameters were calculated using the Pearson test or Spearman rank test, according to the normality of the distribution. To evaluate the effects of continuous variables on gastric cancer staging, multivariate regression analyses were performed using a stepwise selection or enter method. Variables excluded from the initial model were re-entered individually to exclude residual confounding. During development of multivariate regression models, the number of inserted independent variables did not exceed 10% of the total number of analyzed patients. Constructed models were verified using the Akaike information criterion (AIC), and wrongly constructed matrices resulted in rejection of the model. Receiver operating characteristic (ROC) curves were constructed for parameters analyzed as diagnostic for gastric cancer, and the area under each ROC curve (AUC) was calculated. Statistical analysis was performed using SPSS statistical analysis software. P-values less than 0.05 were considered significant.

## Additional Information

**How to cite this article**: Błogowski, W. *et al*. Interleukins 17 and 23 in patients with gastric neoplasms. *Sci. Rep.* **6**, 37451; doi: 10.1038/srep37451 (2016).

**Publisher’s note:** Springer Nature remains neutral with regard to jurisdictional claims in published maps and institutional affiliations.

## Figures and Tables

**Table 1:** General characteristics of analyzed patients and healthy individuals enrolled in the study (data presented as means ± SD or median [interquartile range]).

Parameter	Control	Cancer	Other
Age (years)	61 ± 7	66 ± 11	59 ± 13
Sex (M-male/F-female)	21-M/21-F	25-M/25-F	6-M/19-F
BMI (kg/m2)	25.89 ± 3.29	24.21 ± 3.87	25.56 ± 5.65
RBC (x10^12^ cells/L)	4.79 ± 0.49	4.29 ± 0.92	4.54 ± 0.40
Hb (g/dL)	14.20 ± 1.83	12.50 ± 2.64	13.14 ± 1.55
Platelets count (x10^9^ cells/L)	224 ± 61	265 ± 89	255 ± 91
WBC count (x10^9^ cells/L)	6.07 ± 1.80	6.56 ± 2.28	6.82 ± 2.35
CRP (mg/L)	2.14 ± 1.06	4.13 [1.10; 19.35]	2.45 [1.33; 7.34]

BMI – body mass index, RBC – red blood cells, Hb – hemoglobin, CRP – C-reactive protein, WBC – white blood cells.

**Table 2:** Coefficients of correlations between levels of examined cytokines (IL-17 and IL-23) and both previously reported systemic levels of interleukins together with values of their ratios (n = 46) and absolute numbers of circulating populations of bone marrow-derived stem cells in patients with gastric carcinoma (n = 8).

Parameters/Stem cells population	IL-17	IL-23
**IL-1**	−0.23	−0.24
**IL-6**	−0.11	0.13
**IL-8**	−0.24	−0.11
**IL-10**	−0.01	**−0.39#**
**IL-12**	−0.53*	−0.19
**IL-6/IL-8**	0.05	0.19
**IL-6/IL-10**	−0.01	0.31
**IL-8/IL-10**	0.07	0.20
**VSEL**	0.10	**−0.33#**
**MSC**	0.09	**−0.51#**
**HSC**	−0.03	0.10
**EPC**	0.01	0.07

^#^P < 0.05, *P < 0.005. P – level of significance, IL – interleukin, VSEL – very small embryonic-like stem cells, MSC – mesenchymal stem cells, HSC – hematopoietic stem cells, EPC – endothelial progenitor cells.

**Table 3:** Analysis of associations between levels of examined interleukins and clinical presentation of gastric carcinoma in patients (modelling using multivariate regression analysis).

Dependent variable	Independent variable	β	R^2^	p
Gastric cancer TNM staging*	IL-17	−0.10	0.01	0.48
	IL-23	−0.21	0.05	0.15

β – standardized coefficient in the regression equation. p – level of significance. IL – interleukin. *Variable was created by assigning 1, 2, 3 or 4 value to appropriate TNM stage detected in patients with gastric cancer.
